# A Case of Postablation Pericardial Effusion

**DOI:** 10.19102/icrm.2018.091002

**Published:** 2018-10-15

**Authors:** Jeffrey B. Ziffra, Nicholas D. Germano, Angela N. Phillips, Brian Olshansky

**Affiliations:** ^1^Department of Cardiology, Mercy Medical Center, Mason City, IA, USA; ^2^Department of Medicine, Des Moines University, Des Moines, IA, USA; ^3^Department of Cardiology, University of Iowa, Iowa City, IA, USA

**Keywords:** Atrial fibrillation ablation, intracardiac echocardiography, pericardial effusion

## Abstract

Complications of atrial fibrillation ablation include pericardial effusion, which tends to occur acutely. Large and hemodynamically important effusions are uncommon, but a small effusion may be present at the end of the procedure in up to 22% of ablations. We monitor for pericardial effusions routinely after ablation with intracardiac echocardiography. However, the follow-up of a small effusion present immediately after ablation remains uncertain, especially with the use of dabigatran or another novel oral anticoagulant. There are no current recommendations on the follow-up of small pericardial effusions after ablation. We present a case and ask a panel of experts for their opinions.

## Case presentation

A 74-year-old female with symptomatic, frequent, paroxysmal atrial fibrillation (AF) episodes underwent CARTO^®^ (Biosense Webster, Diamond Bar, CA, USA) map-guided pulmonary vein isolation using a double transseptal technique and a contact force catheter. Oral dabigatran 150 mg (medication typically taken twice daily) was withheld during the previous afternoon and on the morning of the procedure. The patient had a CHA_2_DS_2_-VASc score of 4 and normal renal function. The procedure was performed utilizing standard intravenous heparin anticoagulation with activated clotting time values ranging from 198 seconds to 401 seconds. Heparin was reversed at the end of the procedure with protamine. Intracardiac echocardiography (ICE) was performed before removing the sheaths but after protamine administration and showed a small rim of a pericardial effusion not clearly present at the start of the case. Oral dabigatran was reinitiated that afternoon and continued at a dose of 150 mg once a day for two days after the ablation. The patient was subsequently discharged on a regimen of oral dabigatran 150 mg twice a day, oral colchicine 0.6 mg twice a day, and pantoprazole 40 mg once a day and was scheduled for a follow-up visit.

**Figure 1:**
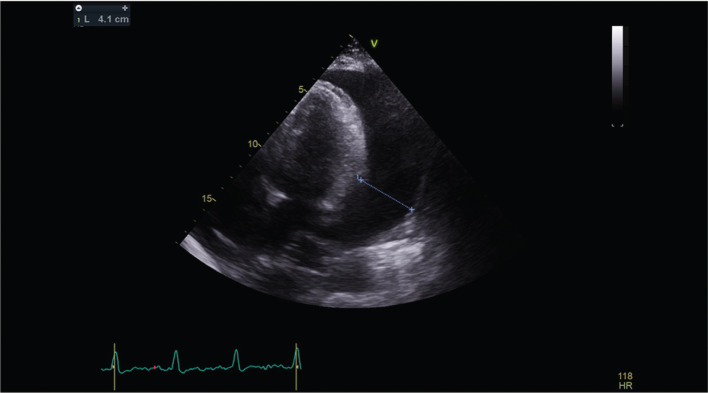
Large 4.1 cm anteroposterior diameter of a posterior pericardial effusion in an apical four-chamber view.

**Figure 2:**
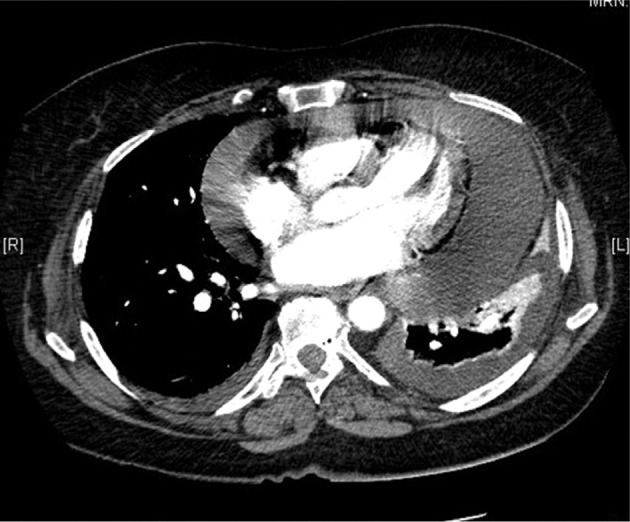
Computed tomography scan revealing a large circumferential pericardial effusion with bilateral pleural effusions.

The patient remained stable without symptomatic AF for a time. However, seven weeks later, just before a follow-up visit, she developed a five-day history of acute and worsening dyspnea. A transthoracic echocardiogram (TTE) revealed a large circumferential pericardial effusion, with the largest diameter being posterior at 4.1 cm, with possible coagulum around the left ventricle **(Figure 1)**. An outpatient computed tomography scan revealed large pericardial and pleural effusions **(Figure 2)**.

The patient was not clinically in cardiac tamponade. A pericardiocentesis yielded 900 cc of red fluid with 1,338,149 red blood cells/μl and 3,746 white blood cells/μl. With drainage, the patient showed modest improvement with respect to the pericardial effusion; after three days, due to decreased output, the drain was subsequently removed. The pleural effusion, which was hazy yellow in color, was also tapped. Both the pericardial and pleural effusions had no growth by culture. The true etiology of both effusions remained unclear. Dabigatran was subsequently discontinued and a repeat TTE performed weeks later revealed resolution of the pericardial and pleural effusions.

## Seeking answers

To our knowledge, no guidelines or recommendations exist on how to manage patients who have a small pericardial effusion seen immediately after a radiofrequency ablation of AF using a double transseptal technique, particularly when a nonvitamin-K oral anticoagulant is used.

In light of this, we recently sought to ask a panel of experts about their experiences in an effort to learn more from our peers. Their responses are available in the accompanying commentaries.

## Discussion

For symptomatic, drug-resistant paroxysmal AF in a septuagenarian, catheter ablation is the standard therapy. Major complications include stroke, pericarditis, cardiac perforation, and cardiac tamponade. Complications are uncommon, with the reported incidence of serious complications amounting to just 6%.^1^ Perforation causing cardiac tamponade is a major concern and is the most common cause of death after the procedure. However, ablations are generally well-tolerated and the mortality risk associated with an AF ablation is only about 0.1%.^2,3^ Small pericardial effusions are more common, but rarely progress.^4^ The follow-up of a patient with a small effusion has not yet been standardized and is concerning in patients prescribed a nonvitamin-K antagonist.

Pericardial effusions are detected by echocardiography, often in an incidental manner, due to their wide range of clinical presentations. Pericardial effusions after AF ablations are not frequently reported, as they are rarely clinically significant. The incidence has been reported to vary between 14.2% and 22%, with greater incidence occurring in patients with coronary artery disease, hypertension, and longer procedure times. Cardiac tamponade, pericarditis, and significant hemorrhagic pericardial effusion are less common postablation.^5^ Intracardiac echocardiography is used for the detection of developing effusions during and immediately after the ablation procedure and is employed routinely in transseptal punctures.^6^

When using a protocol with TTEs performed at 24 hours before, 24 hours after, and one month after the procedure, respectively, up to 22% of patients undergoing ablation had effusions and 89% of these were asymptomatic.^5^ This begs the consideration of whether we should be looking more often or more diligently.

With the implementation of a regular screening protocol, only 3% of ablation patients taking warfarin had a pericardial effusion at five years after ablation.^7^ Patients undergoing ablation of AF had a higher occurrence of pericardial effusion versus those undergoing ablation of atrioventricular nodal reentrant tachycardia or atrial flutter. However, the incidence of significant effusions was still low.^8^ Pericardial effusions that occur late, even at 55 days after ablation, have been reported when rivaroxaban is taken.^9^ In the Study Exploring Two Treatment Strategies in Patients with AF Who Undergo Catheter Ablation Therapy (VENTURE-AF) trial, uninterrupted use of oral rivaroxaban for AF ablation had a similar safety profile as vitamin K antagonist use. This suggests that it is safe to continue the patient on anticoagulation, even during an ablation.^10^

The comparable safety profile of factor Xa inhibitors versus vitamin K antagonists has been well-established. AF ablation on uninterrupted anticoagulation is performed commonly and has demonstrated minimal adverse effects, with little difference between vitamin K antagonists and factor Xa inhibitors.^11–13^ There was little difference between uninterrupted and interrupted apixaban usage as well.^14^ Anticoagulated patients with pericardial effusions after ablation often did well with the resumption of anticoagulation after intervention.^15^ There are no specific treatment recommendations for postablation pericardial effusions, but it may be reasonable to treat with colchicine for a short period of time as is already done for postoperative AF and pericarditis prophylaxis.^16,17^

## Conclusion

We describe a patient who had a small pericardial effusion after ablation that slowly grew into a large effusion over several weeks while she was treated with dabigatran. The cause for the apparent pleural effusion is not entirely known. She was anticoagulated after the ablation per expert consensus recommendations. Small pericardial effusions are not uncommon and can occur in 14.2% or more of ablations. No current recommendations exist on how to monitor these patients or regarding whether anticoagulation should be curtailed or modified if a small effusion is present or not. The cause of the pericardial and pleural effusions in this case is not known. It is possible that the transudative pleural effusion was due to an inflammatory pleuropericarditis that developed subsequent to the ablation, as no perforation was discovered during the procedure. There have been reports for treatment of this syndrome with colchicine.^18^ We appreciate the input of our peers regarding this case and hope that our experience and their opinions will serve to assist others facing similar cases.

## References

[1] Cappato R, Calkins H, Chen SA (2005). Worldwide survey on the methods, efficacy, and safety of catheter ablation for human atrial fibrillation. Circulation.

[2] Cappato R, Calkins H, Chen SA (2009). Prevalence and causes of fatal outcome in catheter ablation of atrial fibrillation. J Am Coll Cardiol..

[3] Calkins H, Kuck KH, Cappato R (2012). 2012 HRS/EHRA/ECAS expert consensus statement on catheter and surgical ablation of atrial fibrillation: recommendations for patient selection, procedural techniques, patient management and follow-up, definitions, endpoints, and research trial design: a report of the Heart Rhythm Society (HRS) Task Force on Catheter and Surgical Ablation of Atrial Fibrillation. Developed in partnership with the European Heart Rhythm Association (EHRA), a registered branch of the European Society of Cardiology (ESC) and the European Cardiac Arrhythmia Society (ECAS); and in collaboration with the American College of Cardiology (ACC), American Heart Association (AHA), the Asia Pacific Heart Rhythm Society (APHRS), and the Society of Thoracic Surgeons (STS). Endorsed by the governing bodies of the American College of Cardiology Foundation, the American Heart Association, the European Cardiac Arrhythmia Society, the European Heart Rhythm Association, the Society of Thoracic Surgeons, the Asia Pacific Heart Rhythm Society, and the Heart Rhythm Society.. Heart Rhythm..

[4] Chierchia GB, Capulzini L, Droogmans S (2010). Pericardial effusion in atrial fibrillation ablation: a comparison between cryoballoon and radiofrequency pulmonary vein isolation. Europace.

[5] Lellouche N, Sebag FA, Elbaz N (2011). Acute pericardial effusion following atrial fibrillation ablation: characteristics and relationship with arrhythmia recurrences. Arch Cardiovasc Dis..

[6] Cooper JM, Epstein LM (2001). Use of intracardiac echocardiography to guide ablation of atrial fibrillation. Circulation.

[7] Weerasooriya R, Khairy P, Litalien J (2011). Catheter ablation for atrial fibrillation: are results maintained at 5 years of follow-up?. J Am Coll Cardiol..

[8] Schaer BA, Maurer A, Sticherling C, Buser PT, Osswald S (2009). Routine echocardiography after radiofrequency ablation: to flog a dead horse?. Europace.

[9] Kitamura T, Fukamizu S, Sakurada H, Hiraoka M (2014). Development of delayed cardiac tamponade 55 days after catheter ablation for atrial fibrillation with a new oral anticoagulant. J Interv Card Electrophysiol..

[10] Lakkireddy D, Reddy YM, Di Biase L (2014). Feasibility and safety of uninterrupted rivaroxaban for periprocedural anticoagulation in patients undergoing radiofrequency ablation for atrial fibrillation: results from a multicenter prospective registry. J Am Coll Cardiol..

[11] Garg J, Chaudhary R, Krishnamoorthy P (2016). Safety and efficacy of oral factor-Xa inhibitors versus vitamin K antagonist in patients with non-valvular atrial fibrillation: meta-analysis of phase II and III randomized controlled trials. Int J Cardiol..

[12] Dillier R, Ammar S, Hessling G (2014). Safety of continuous periprocedural rivaroxaban for patients undergoing left atrial catheter ablation procedures. Circ Arrhythm Electrophysiol..

[13] Ukaigwe A, Shrestha P, Karmacharya P (2017). Meta-analysis of efficacy and safety of apixaban and uninterrupted apixaban therapy compared to vitamin K antagonists in patients undergoing catheter ablation for atrial fibrillation. J Interv Card Electrophysiol..

[14] Garg J, Chaudhary R, Krishnamoorthy P, Shah N, Bozorgnia B, Natale A (2016). Safety and efficacy of uninterrupted periprocedural apixaban in patients undergoing atrial fibrillation catheter ablation: a metaanalysis of 1,057 patients. J Atr Fibrillation.

[15] Gianni C, DI Biase L, Mohanty S (2016). Management of periprocedural and early pericardial effusions with tamponade following ablation of atrial fibrillation with uninterrupted factor Xa inhibitors: a case series. J Cardiovasc Electrophysiol..

[16] Deftereos S, Giannopoulos G, Efremidis M (2014). Colchicine for prevention of atrial fibrillation recurrence after pulmonary vein isolation: mid-term efficacy and effect on quality of life. Heart Rhythm..

[17] Imazio M, Brucato A, Ferrazzi P (2011). Colchicine reduces postoperative atrial fibrillation: results of the Colchicine for the Prevention of the Postpericardiotomy Syndrome (COPPS) atrial fibrillation substudy. Circulation.

[18] Yukumi S, Ichiki H, Funada J (2015). Postcardiac injury syndrome following vascular interventional radiofrequency ablation for paroxysmal atrial fibrillation. Respir Med Case Rep..

